# A rapid, convenient, solventless green approach for the synthesis of oximes using grindstone chemistry

**DOI:** 10.1186/2191-2858-1-12

**Published:** 2011-10-04

**Authors:** Lakhinath Saikia, Jejiron Maheswari Baruah, Ashim Jyoti Thakur

**Affiliations:** 1 Department of Chemical Sciences, Central University, Tezpur, Napaam, Tezpur 784028, Assam, India; 2 Chembiotek, Kolkata, India, C/o TCG Lifesciences Ltd., Block BN, Sector V, Salt Lake City, Kolkata 700 091, India

**Keywords:** oximes, carbonyl compounds, Bi_2_O_3_, grindstone chemistry, solventless, eco-friendly

## Abstract

**Background:**

Synthesis of oximes is an important reaction in organic chemistry, because these versatile oximes are used for protection, purification, and characterization of carbonyl compounds. Nitriles, amides via Beckmann rearrangement, nitro compounds, nitrones, amines, and azaheterocycles can be synthesised from oximes. They also find applications for selective *α*-activation. In inorganic chemistry, oximes act as a versatile ligand.

Several procedures for the preparation of oximes exist, but, most of them have not addressed the green chemistry issue. They are associated with generation of pollutants, requirement of high reaction temperature, low yields, lack of a generalized procedure, etc. Hence, there is a demand for developing an efficient, convenient, and non-polluting or less polluting alternative method for the preparation of oximes. In this context, bismuth compounds are very useful as they are cheap in general, commercially available, air stable crystalline solids, safe, and non-toxic, hence easy to handle.

**Results:**

Carbonyl compounds (aliphatic, heterocyclic, and aromatic) were converted into the corresponding oximes in excellent yields by simply grinding the reactants at room temperature without using any solvent in the presence of Bi_2_O_3_. Most importantly, this method minimizes waste disposal problems, provides a simple yet efficient example of unconventional methodology and requires short time.

**Conclusions:**

We have developed a novel, quick, environmentally safe, and clean synthesis of aldoximes and ketoximes under solvent-free grinding condition.

## 1. Background

Conversion of carbonyl functionalities into oximes is an important reaction in organic chemistry. Oximes are highly crystalline compounds that find applications not only for protection, but also for purification and characterization of carbonyl compounds [1,2].Conversions into nitriles [3], nitro compounds [4,5], nitrones [6], amines [7], and synthesis of azaheterocycles [8] are some of the synthetic applications of oximes. They are also useful for selective *α*-activation [9] and are extensively used as intermediates for the preparation of amides by the Beckmann rearrangement [10,11] and fungicides and herbicides [12]. In inorganic chemistry, oximes act as a versatile ligand.

Classically, oximes are prepared [2] by refluxing an alcoholic solution of a carbonyl compound with hydroxylamine hydrochloride and pyridine. The method has multiple drawbacks such as low yields, long reaction time, toxicity of pyridine, and effluent pollution caused by the use of organic solvent. In recent times, solvent-free reactions have drawn considerable attention and popularity [13,14], not only from an environmental point of view, but also for synthetic advantages in terms of yield, selectivity, and simplicity of the reaction procedure. Since chemical industry deals with larger quantity of materials, these factors are particularly very important therein. Over the years, many reagents and catalysts have been developed for the synthesis of oximes. Basic aluminia [15], CaO [16], and TiO_2_/(SO_4_^2-^) [17] coupled with microwave irradiation under solvent-free condition have been claimed to be efficient methods for the preparation of oximes. Hashem Sharghi and Hosseini [18] described a solventless reaction protocol for synthesizing aldoximes from corresponding aldehydes using ZnO as catalyst at 80°C. Interestingly, they obtained Beckmann rearrangement product at higher temperatures (140-170°C). More recently, conversion of carbonyl compounds to oximes in aqueous biphasic medium and ionic liquid/water biphasic system [19,20] has been reported. However, problems of generation of polluting HCl, high reaction temperature, occasionally low yields, and lack of a generalized procedure covering all types of aldehydes and ketones still present. Consequently, there is a demand for developing an efficient, convenient, and non-polluting or less polluting alternative method for the preparation of oximes. In this context, because of the rich chemistry of bismuth compounds [21-25], we became interested therein. Bismuth compounds are generally cheap, commercially available, air stable crystalline solids, safe, and non-toxic, hence easy to handle. Their Lewis acidity is also well known [26,27]. Most bismuth(III) compounds have an LD_50 _value which is comparable to or even less than that of NaCl [28].

**Scheme 1:**
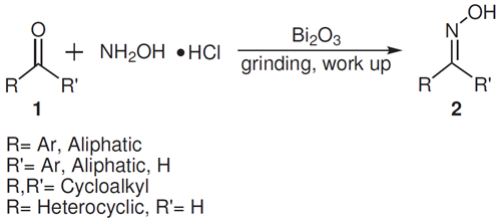
**Synthesis of aldoximes and ketoximes**.

In continuation to our interest in protection and deprotection chemistry [29,30], we have developed a novel, quick, environmentally safe, and clean synthesis of aldoximes and ketoximes under grinding condition (Scheme 1) utilizing pestle and mortar under solvent-free condition. The method makes use of local heat generated by simply grinding the reactants and catalyzed by cheap and commercially available Bi_2_O_3 _for driving the chemical reaction at room temperature. The work up is easy and furnished the oximes in excellent yields. Most importantly, this method minimizes waste disposal problems and provides a simple yet efficient example of unconventional methodology, which is equally effective for all types of aldehydes and ketones. Earlier reports [15,31] of similar type restricted their utility in carbonyl compounds (alicyclic and aliphatic) and aromatic aldehydes only and aromatic ketoximes were not obtained. In those reports, for ketoxime synthesis, microwave irradiation or addition of some other additives was necessary. In this regard, our method is superior, quite general, and versatile.

## 2. Results and discussion

**Figure 1:**
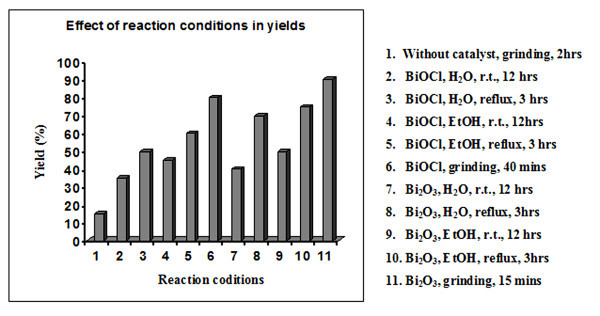
**Effect of reaction conditions in yields**.

In the present solvent-free method, the effectiveness of Bi_2_O_3 _in oxime synthesis (see Scheme 1) under grinding condition is demonstrated using a broad spectrum of aldehydes and ketones with hydroxylamine hydrochloride in the absence of a base or any other additives. To search for the best reaction condition for oximation using easily available bismuth compounds, a set of reactions have been carried out using *p-*chlorobenzaldehyde and hydroxylamine hydrochloride as substrates under various reaction conditions at constant catalyst (Bi_2_O_3 _and BiOCl) loading (50 mol% with respect to substrate). Figure 1 summarizes the results, which clearly shows that the Bi_2_O_3 _under solvent-free grinding condition is the most effective.

After surveying a series of reaction conditions, the optimized results are summarized in Table 1. Aromatic, aliphatic, heterocyclic, and *α*,*β*-unsaturated aldehydes were converted to the corresponding oximes in almost quantitative yields within very short time (1.5-3 min, entries 1a-k, Table 1). Cinnamaldehyde (entry 1j, Table 1) was smoothly converted to cinnamaldehyde oxime without any rearrangement of *α*,*β*-double bond. For ketones (acyclic and cyclic), however, reactions were comparatively difficult and took a little longer time (5.5-20 min, entries 1l-q, Table 1). It was interesting to note that the less reactive benzophenone also condensed with hydroxylamine hydrochloride in 60% yield only that too requiring longer time, i.e., 20 min (entry 1q, Table 1). 5α-Pregn-16-en-3α-ol-20-one acetate oxime (entry 1l, Table 1) was obtained in 88% yield within 6 min. The unreacted materials were recovered from the reaction mixture. No observable difference in reactivity exerted by -NO_2 _group at *m*- or *p*-position was noticed (entries 1b and c, Table 1), being yields and reaction times were almost same. Entities such as chloro, nitro, hydroxyl were found to be inert to the reaction condition. The products were identified by their spectral (^1^H NMR,^13^C NMR, IR spectra) data, physical data (melting point, elemental analysis), and comparison with authentic ones. IR spectra supported this observation as no peak was observed around 2200 cm^-1 ^characteristic of the -C≡N group. However, appearance of peaks around 3200-3450 and 1600-1680 cm^-1 ^are indicative of -OH and >C = N- groups, respectively. In^1^H NMR spectra, the -OH signal of oximes appeared within δ = 8.0-10.00 ppm as a broad singlet (characteristic signal) that was exchangeable with D_2_O. It was very appealing that in these reactions neither the dehydration product, nitriles, nor the amides, via Beckmann rearrangement were observed. The reaction was very clean and no other product was observed.

**Table 1:** Preparation of aldoximes and ketoximes 2a

Entry	Substrate 1	Product 2	Time (min)	Isolated yield (%)	Mp/°C (Lit.)
a			1.5	95	-(35)^b^
b			2	96	105 (107)^b^
c			2	96	130 (133)^b^
d			2	95	120 (122)^b^
e			2	>98	75 (80)^b^
f			3	95	74 (72)^b^
g			2.5	98	128 (132)^b^
h			2	95	72 (-)^c^
i			2	>98	128 (132-136)^b^
j			2	>98	135 (139)^b^
k			6	88	197 (195.5-198)^b^
l			3	96	-(52)^b^
m			5.5	92	-(57)^b^
n			6	95	86 (91)^b^
o			7	>98	56 (59)^b^
p			6.5	>98	86 (88)^b^
q			20	60	140 (144)^b^

^a^All compounds were characterized on the basis of IR,^1^H and^13^C NMR spectral data, Mass spectrometry data and m_p_.

^b^(i) Furniss BS, Hannaford AJ, Smith PWG, Tatchell AR (2008) Vogel's textbook of practical organic chemistry, 5th edn. Pearson Education, Dorling Kindersley (India). (ii) Alfa-Aesar Research Chemicals, Metals and Materials catalogue, 2008-2009.

^c^Not found in the literature.

To evaluate the synergy between rate, yield, and Bi_2_O_3 _loading in this reaction, several experiments were carried out. In a pilot experiment, the reaction was found to proceed poorly in the absence of Bi_2_O_3_. As far as Bi_2_O_3 _loading is concerned, 60 mol% of the catalyst with respect to the substrate was the optimum one (Table 2). An increase in Bi_2_O_3 _loading did not improve the yield as well as no change in reaction time was observed. However, a decrease in Bi_2_O_3 _loading appreciably decreased the rate and yield of the reaction.

**Table 2:** Optimization of catalyst loading in preparation of 2b

Entry	Catalyst loading (mol%)	Time	Yield (%)
1	No	2 h	15
2	20	30 min	48
3	30	30 min	57
4	40	30 min	70
5	50	15 min	90
6	60	2 min	96
7	70	2 mins	96

We have also checked the reusability of the catalyst using the recovered Bi_2_O_3 _from the reaction. It is observed that recovered catalyst could be satisfactorily used for the second run, whereas, third run of the recovered catalyst leads to poor yield and longer reaction time (Table 3). The surface areas of the fresh as well as the recovered catalyst after the third run in the reaction were determined in a surface area and pore size analyzer and found to be 5.21 and 37.106 m^2^/g, respectively. The average particle diameters of the fresh as well as the recovered catalyst after the third run in the reaction were calculated out from these measured surface areas and were found to be 129.396 and 18.168 nm, respectively. The increase in granularity of the catalyst after reuse is obvious since it was grounded. However, the decrease in efficiency of the catalyst after the third run might be due to the loss of active sites of the catalyst.

**Table 3:** Reusability of Bi_2_O_3 _in the preparation of 2b using 60 mol% of the catalyst

Entry	Run number	Time	Yield (%)
1	1	2	96
2	2	8	87
3	3	20	52

## 3. Conclusions

To the best of our knowledge, Bi_2_O_3 _has never been used in the synthesis of oximes earlier. In conclusion, the reported procedure is an interesting, extremely simple, suitable, fast, efficient, and novel method for the preparation of oximes. The methodology also offers chemical, economical, and environmental advantages. On the other hand, Bi_2_O_3 _is remarkably easier to use, non-hazardous, inexpensive and work under mild neutral conditions [32,33].

## 4. Experimental

Melting points were determined on a Büchi 504 apparatus and were uncorrected. IR spectra were recorded in KBr pallets on a Nicolet (Impact 410) FT-IR spectrophotometer.^1^H NMR and^13^C NMR spectra were recorded on a JNM ECS 400 MHz FT-NMR (JEOL) spectrophotometer with TMS as the internal standard. Mass spectra were recorded on a Waters Q-TOF Premier & Aquity UPLC spectrometer. Surface area of the catalyst before and after use in the reaction was measured using surface area & pore size analyzer (NOVA 1000e, Quanta chrome Instruments). All the chemicals were used as-received.

## 5. Methods

### 5.1. Typical procedure for the formation of oxime 2

A mixture of aldehyde/ketone **1 **(1 mmol), hydroxylamine hydrochloride (1.2 mmol), and Bi_2_O_3 _(0.6 mmol) was grounded in a mortar with a pestle for the required period of time. On completion of the reaction as monitored by TLC, ethyl acetate (2 × 10 mL) was added to the reaction mixture and filtered to separate the Bi_2_O_3_. The filtrate was concentrated down to approx. 6 mL, then added water to it when product precipitated out from the solution. The precipitate was filtered out and dried in high vacuum to furnish the pure oxime **2 **in 60-98% yield.

## Abbreviations

IR: infrared; LD_50_: lethal dose that kills half (50%) of the animals tested; NMR: nuclear magnetic resonance.

## Competing interests

The authors declare that they have no competing interests.
